# Oral Cancer: Prophylaxis, Etiopathogenesis and Treatment

**DOI:** 10.3390/cimb46110768

**Published:** 2024-11-13

**Authors:** Violeta Popovici, Emma Adriana Ozon

**Affiliations:** 1 Center for Mountain Economics, “Costin C. Kiriţescu” National Institute of Economic Research (INCE-CEMONT), Romanian Academy, 725700 Vatra-Dornei, Romania; 2 Department of Pharmaceutical Technology and Biopharmacy, Faculty of Pharmacy, “Carol Davila” University of Medicine and Pharmacy, 020956 Bucharest, Romania; emma.budura@umfcd.ro

Oral cancer contributes to approximately 3–10% of all cancer mortality worldwide, and its incidence is continuously increasing due to environmental conditions and harmful habits of the modern lifestyle [1]. In association with hereditary predisposition, chronic inflammation, and infectious diseases, these factors raise the frequency of oral cavity malignancies. Major intervention involves extensive surgery associated with radio and chemotherapy [1]. Moreover, advancements in cancer immunotherapy have not yet established the specific mechanisms by which immune cells influence tumor progression and immune evasion, and oral cancers continue to inadequately respond to the current treatment protocols. The abovementioned aspects underline this Special Issue, which has brought together the most recent research on molecular mechanisms implied in prophylaxis, etiopathogenesis, and the treatment of oral cancer. New and valuable data have been added to this field by the authors of the 10 articles contained in this publications: nine original papers and one review.

Oral squamous cell carcinoma (OSCC) represents 90% of oral neoplasias; it is the sixth most frequent malignancy in the world, with an overall 5-year survival rate below 50% due to its modest outcomes, tardive diagnosis, aggressive local invasiveness, recurrences, and metastases. OSCC belongs to the comprehensive group of head and neck squamous cell carcinomas (HNSCCs), which affects the cell lining of the oral cavity, pharynx, and larynx. The local–regional recurrence rate for advanced carcinomas is over 50% despite administration of multimodal therapy, and understanding the biology of HNSCCs is essential to ameliorate their prognosis [1].

Thus, the authors of the first contribution integrated the cells isolated from a hypopharyngeal tumor of a squamous cell carcinoma patient (FaDu) in an oral mucosa model (OMM) obtained by seeding primary human fibroblasts and keratinocytes onto a porcine small intestinal submucosa with preserved mucosa (SIS/MUC). They generated a 3D model that mimics the crosstalk at the tumor front of human HNSCC by enabling cellular and stromal interactions and revealing the features of tumor cell invasiveness [1]. Their work created the premise of integrating further tumor microenvironment components to establish the molecular mechanisms of the most effective anticancer therapy.

One of the most important components of stromal cells is tumor-associated macrophages, which are related to poor prognoses. The second contribution suggests that M2 macrophage-derived exosomes could induce OSCC cell proliferation, invasion, and migration and inhibit tumor cell apoptosis by transferring miRNA-23a-3p into tumor cells [2]. Additionally, the authors revealed that phosphatase and tensin homolog (PTEN), a well-known tumor-suppressor gene, could be a potential cellular target for miRNA-23a-3p to promote OSCC development.

In a retrospective study, Takasaki et al. examined the surgically resected tissues of 70 patients with oral potentially malignant disorders (OPMDs) via immunochemistry and statistically analyzed the association of the target proteins’ (p53, p62, Ki67, and XPO1) expressions with intracellular distribution, malignant transformation, and clinicopathological characteristics [3]. They found that Ki67, a well-known marker of cell proliferation shows a significant positive correlation with p62 expression in the cell cytoplasm and aggregation expression and a negative one with p62 expression in the nucleus. This third contribution suggests that the autophagy-related multidomain protein p62 could be a potential biomarker for the risk of the neoplastic transformation of OPMDs [3].

Using human in vitro and ex vivo models of oral dysplasia from the tongue, Peña-Oyarzún et al. found that 1,25-(OH)2D3 increased nuclear vitamin D receptors (VDR) and membranous expression of E-cadherin and diminished the Ki67 expression and nuclear localization of β-catenin [4]. Thus, this fourth contribution shows that 0.1 µM Calcitriol treatment could diminish the risk of malignant transformation of OPMDs by reducing cell proliferation, migration, and β-catenin signaling [4].

Many types of cancer alter the energetic metabolism, thus decreasing oxidative processes and raising glucose uptake and glycolysis. Glycolytic activity significantly increases the development of HNSCCs, and this particularity could be considered a potential therapeutic target. Therefore, in the fifth contribution, Kleszcz et al. show that the expression of glycolytic enzymes in tongue squamous cell carcinoma could be modulated by the Wnt signaling pathway inhibitors PRI-724 and IWP-O1 [5].

MicroRNAs (miRNAs) are endogenous small RNA molecules that are single-stranded and non-coding; they circulate in a stable form and display aberrant expression in various malignancies. Differentially expressed miRNAs could be potential biomarkers for cancer screening. Moreover, next-generation cancer therapy could be based on miRNA modulation. In the sixth contribution, the authors identified a five-miRNA diagnostic model associated with tongue squamous cell carcinoma patients. They highlighted the remarkable diagnostic potential of miR-196b and selected five hub genes from the target ones of miR-196b [6].

CD44 is a transmembrane protein with important roles in cell proliferation, adhesion, migration, and lymphocyte activation. Various types of CD44 are expressed in cancer cells, especially in the advanced stages, and their expression is detected using monoclonal antibodies. In the seventh contribution, Ishikawa et al. analyzed C44Mab-18 (a novel anti-CD44 variant with 10 monoclonal antibodies) for immunohistochemical analysis of oral squamous cell carcinomas [7].

Recent studies investigating genome alteration in HNSCC patients have attracted wide attention [8]. The eighth contribution of this Special Issue describes novel mutations in MUC6 and MUC16, providing new insight into the genetic alternation in mucin genes among oropharyngeal squamous cell carcinoma (OPSCC) patients. This research could initiate further studies, including larger cohorts, to recognize the pattern in which the mutations affect oropharyngeal carcinogenesis.

Understanding the molecular mechanisms implied in the development and progression of OSCC is essential for improving diagnostic and therapeutic strategies. Considering oral squamous cell carcinomas in dogs as an excellent model for studying human counterparts, Files et al. investigated the significance of two key molecular components, Cox-2 and EGFR, in canine OSCC. Their findings revealed that Cox-2 was highly expressed in 70.6% of cases, while EGFR overexpression was observed in 44.1%; Cox-2 overexpression was associated with the histological grade of malignancy (HGM) and EGFR with vascular invasion. Therefore, the results of this ninth contribution suggest that Cox-2 and EGFR could be promising biomarkers and potential therapeutic targets, leading to the development of novel treatment strategies for OSCC therapy.

Analyzing the major risk factors for HNSCC, the tenth contribution highlights that human papillomavirus (HPV) is correlated with a high incidence of oropharyngeal cancers. Moreover, the accessed literature data show that HPV vaccines approved for cervical cancer prevention in females had a notable impact on HNSCC incidence [9]. In addition, this comprehensive review investigates various mechanisms of inducing immunogenicity against HNSCC cells, including traditional approaches (cell-mediated cytotoxicity induced by antigens), as well as innovative strategies (to counteract tumor immune escape mechanisms or stimulate the immune system’s cytotoxic activity against neoplastic cells). The last three contributions are listed below.
